# Functional connectivity gradients of the insula in major depressive disorder

**DOI:** 10.3389/fpsyt.2026.1792843

**Published:** 2026-04-15

**Authors:** Birce Begum Burhanoglu, Ozgul Uslu, Aslihan Uyar, Ali Saffet Gonul

**Affiliations:** 1 Standardization of Computational Anatomy Techniques for Cognitive and Behavioral Sciences (SoCAT) Lab, Department of Psychiatry, School of Medicine, Ege University, Izmir, Türkiye; 2 Department of Neuroscience, Health Sciences Institute, Ege University, Izmir, Türkiye; 3 Department of Psychiatry, Mugla Sıtkı Kocman University Training and Research Hospital, Mugla, Türkiye

**Keywords:** functional connectivity, gradient analysis, insula, major depressive disorder, network connectivity

## Abstract

**Introduction:**

The insula, as a key component of the salience network, plays a crucial role in cognition, emotion regulation, and interoception. Functional abnormalities in the insula have been consistently reported in patients with major depressive disorder (MDD). While prior research has examined functional connectivity gradients in MDD at the whole-brain and network levels, and has characterized insular gradient organization in healthy individuals and related affective disorders, the specific topology of insula functional connectivity gradients in MDD has not yet been directly investigated. This study aimed to reveal the differences in gradient properties of the insula between MDD patients and healthy controls.

**Methods:**

Resting-state functional MRI data were collected from 38 MDD patients and 34 age- and gender-matched healthy controls. Functional connectivity gradients were analyzed at three levels: whole-brain, insula-to-whole-brain, and insula-to-seven-brain-networks.

**Results:**

At the whole-brain level, the principal gradient distinguished transmodal regions from unimodal regions in both groups. However, the insula-to-whole-brain gradient axis demonstrated a less distinct separation between anterior and posterior insular regions in MDD patients. Additionally, the insula’s functional relationship with the dorsal attention network exhibited greater specialization in MDD patients compared to healthy controls.

**Discussion:**

These findings extend existing research on insular functional connectivity in depression, highlighting altered gradient properties in MDD. Future research may focus on the relationship between dorsal attention network and insula while taking the laterality of gradients into consideration and assess the reproducibility of the findings.

## 1 Introduction

Major depressive disorder (MDD) is one of the most prevalent psychiatric diseases and a leading cause of disability worldwide. An estimated 4.4% of the world’s population is diagnosed with depression, which corresponds to a population of over 300 million (1). Despite its prevalence, the neurobiological underpinnings of MDD remain partially understood, negatively impacting the development of effective prevention and treatment strategies. Therefore, identifying neural organization patterns that distinguish patients from healthy individuals remains a critical research priority.

Recent advances in neuroimaging have introduced gradient analysis as a framework for characterizing the brain’s hierarchical organization. This approach offers a powerful alternative to traditional parcellation, moving beyond discrete regional boundaries to capture the continuous nature of functional specialization (2, 3). It identifies the main variance axes of brain data through dimensionality reduction techniques (e.g. diffusion map embedding) and simplifies the interpretation of these data by replacing their essentially numerous dimensions with fewer dimensions (i.e. gradients) that explain most of the variance. Each gradient is a continuous representation of an aspect of brain organization, and accordingly, each brain region can be assigned a value that reflects where it falls on this continuum. Along these few-dimensional axes (i.e. functional gradients), brain regions that share many strong neural connections, or a few very powerful connections, are positioned close together. Conversely, areas with weak or no connections are placed farther apart. Unlike traditional parcellation or seed-based connectivity analyses, gradient analysis captures the global or regional topology of connectivity transitions, offering a better representation of functional integration and segregation (2). Moreover, studies report replicable gradient features across independent cohorts and improved prediction accuracy of symptoms with connectivity gradients compared to traditional connectivity measures (4, 5), making the gradient approach a promising tool for identifying neurobiological differences in psychiatric research.

Previous research in healthy populations showed that a functional connectivity gradient distinguishes primary sensorimotor areas from higher-order association regions, while gradients based on cortical microstructure differentiates sensory/motor areas from paralimbic regions (2, 3). These normative findings have been interpreted as reflecting large-scale principles underlying motor control, cognition, and emotion. They provide a spatial benchmark, so that a significant shift in a region’s position along those gradients may indicate a disruption in the hierarchical processing of information. While initial gradient research was on healthy individuals to establish these baselines, recent psychiatric research revealed functional gradient alterations at the whole-brain or network levels in disorders such as schizophrenia and autism spectrum which were correlated with symptom severity (6, 7). In MDD, only a few studies have investigated whether the functional gradient structure is disrupted in MDD patients and whether this disruption is related to other variables associated with the disease (8–16). These MDD studies mostly focused on the whole-brain or specific networks in terms of gradient organization. In contrast, studies with healthy populations have discovered specific brain regions’ functional gradients such as precuneus, thalamus, angular gyrus, cingulate cortex, corpus callosum and insula (17–22). Therefore, these region-specific studies are lacking for MDD populations except for one recent study that focused on limbic, thalamic and basal ganglia system functional gradients (23).

The insula is a critical region of interest for such region-specific studies in depression. It is a multifunctional region located just below the lateral sulcus and has connections to many different nearby/distant brain regions. It shows a posterior-to-anterior hierarchy that reflects differences in the types of inputs it receives and its topographical connections with other brain regions. Posterior and middle insula regions receive sensory and interoceptive inputs mainly via the thalamus, and are strongly connected with sensory and motor cortices. In contrast, the ventral anterior insula receives interoceptive inputs through several subcortical and cortical pathways and shows stronger connections with prefrontal regions. Accordingly, the anterior insula has a role in emotional awareness, interoception and cognition, the posterior insula has a role in somatosensory processing, and the middle insula integrates information from posterior sensory representations with anterior higher-order processes (24–27). Within an active inference framework, the brain continuously generates top-down interoceptive predictions to explain ascending sensory data from visceral organs, seeking to minimize prediction errors (the difference between predicted and actual states). These processes are organized across the insula’s hierarchical architecture. Moreover, proper functioning of these processes are considered to be related with protecting mental health (28). Therefore, the insula is positioned as a key brain region that processes what is happening inside the body, helping shape our behavior and our subjective feeling states. In MDD, the insula exhibits different functional connectivity patterns with the default mode, frontoparietal and salience networks (29–32). Besides, the connectivity of the insula, especially its anterior region, has a predictive role in response and non-response to treatment methods such as medication, psychotherapy and TMS (33–36). While these studies highlight the insula as an important hub in MDD-related network dysfunction, they do not capture the internal transitions within the insula itself or how it is positioned within the brain network organization.

Gradient studies in healthy individuals provide a normative reference for addressing this gap. Previous research revealed that the insula’s functional connectivity is best modeled as a continuum of gradual change from dorsal-posterior to ventral-anterior, i.e., on a gradient axis. This functional differentiation across the region was also linked to individual variations in positive affect, self-efficacy, emotion recognition and motor skills (37). Additional gradients representing insular regions’ differentiated functional relationship with unimodal cortical regions, transmodal association areas and subcortical structures were also captured in a healthy sample (21). A single exploratory study found that individuals with a cognitive vulnerability to depression showed altered gradient scores in the ventral insula, which were associated with hopelessness and rumination (10). However, insular gradient organization in MDD, whether its internal hierarchical differentiation deviates from healthy controls remains to be examined.

Based on these evidence of a dorsal–posterior to ventral–anterior insular functional axis in healthy individuals (21, 37), prior reports of altered anterior insular connectivity in MDD (33–36), and the altered gradient structure in the insula among individuals with a cognitive vulnerability to depression (10), we hypothesized that MDD patients would exhibit anterior–posterior insula differentiation reflected by the altered positioning on the whole-brain and on network gradients. Therefore, this study aims to reveal the differences in gradient properties of the insula between MDD patients and healthy controls.

## 2 Materials and methods

### 2.1 Participants

After the Institutional Ethics Committee for Medical Studies approved the study in accordance with the provisions of the Declaration of Helsinki (approval date & number: 4 March 2021, (21-3T/35) 24-3T/88), we recruited individuals between the ages of 18–60 diagnosed with MDD (n=44) and age and gender matched healthy individuals (n=39). All participants signed an informed consent. A psychiatrist (A.U.) confirmed the diagnosis of the MDD group via SCID-I and evaluated the current severity of the symptoms with the Hamilton Depression Rating Scale-17 items (HAM-D).

Inclusion criteria for MDD patients were as follows: 1) being between the ages of 18-60, 2) completion of at least eight years of education, 3) diagnosis of MDD based on DSM-5 criteria, with clinical stability over the past three months (i.e., no symptom changes requiring interventions such as medication adjustments or hospitalization), 4) a score of 19 or above on the HAM-D scale, 5) absence of any other axis I diagnosis, 6) right-handedness. Inclusion criteria for HC are as follows: 1) being between the ages of 18-60, 2) completion of at least eight years of education, 2) absence of any other axis I diagnosis, 3) right-handedness. The exclusion criteria for both groups are as follows: 1) presence of diabetes, uncontrolled hypertension and other cardiovascular diseases, 2) structural MRI revealing significant morphological anomalies, 3) any condition that contraindicates MRI scanning (pacemaker, prosthesis, claustrophobia, etc.).

Six individuals from the depression group (one due to structural MRI pathology, one due to head movement, one due to low image quality, and three due to failure to complete the scan) and five individuals from the healthy control group (HC) (one due to head movement, two due to low image quality, two outliers due to low correlation values with the template values after gradient alignment) were excluded. We used 38 MDD group participants’ and 34 HC group participants’ data for further analysis.

### 2.2 MRI acquisition

MRI scanning was performed using a Siemens Magnetom Verio, Numaris/4, Syngo MR B17 MR scanner with a 12-channel head coil (Erlangen, Germany) in the 3 Tesla MR unit of the hospital. First, T2-weighted axial TSE and coronal 3D-SPACE FLAIR (Dark Fluid) were obtained to detect any pathology, and T1-weighted 3D-MP-RAGE sequence (repetition time (TR) = 1600 ms, echo time (TE) = 2.21 ms, flip angle (FA) = 9°, matrix size 256 x 256, voxel size 0.5 x 0.5 x 1 mm, slice thickness 1 mm, 160 axial slices) were obtained for structural images. Functional images were then obtained using a T2*-weighted Echo-planar imaging (EPI) sequence (TR = 3000 ms, TE = 30 ms, FA = 90°, matrix size 64 x 64, voxel size 3 x 3 x 3.75 mm, slice thickness 3 mm, 42 axial slices). Functional resting state (eyes-closed) acquisition lasted 8 minutes and 160 MRI images were obtained for each participant.

### 2.3 fMRI data processing

#### 2.3.1 Preprocessing

Firstly, the raw fMRI data of the participants in DICOM data format were converted to NIFTI data format in accordance with the Brain Imaging Data Structure (BIDS) format using dcm2bids software (38). Structural images were segmented into white matter, gray matter, and cerebrospinal fluid; then functional images were subjected to standard preprocessing steps using fMRIPrep software version 23.2.2 (39). See the **Supplementary Materials** for preprocessing details. Motion-contaminated volumes were identified using FD > 0.2 mm or standardized DVARS > 3 thresholds. Individual preprocessing reports generated by fMRIPrep, including FD and DVARS time series plots, carpet plots were visually inspected for all participants to ensure adequate data quality and to confirm the absence of excessive motion or preprocessing artifacts by B.B.B. and O.U.

#### 2.3.2 Parcellation and connectivity matrices

Brain parcellation and calculation of connectivity matrices were performed using nilearn (https://nilearn.github.io/stable/index.html). For each participant, parcellation was done using the Schaefer-Yeo atlas, which is a functional cortical parcellation that divides 400 cortical regions of interest (ROI) into seven brain networks [visual (VN), somatomotor (SMN), dorsal attention (DAN), salience/ventral attention (SN), limbic (LN), control (CN), default mode (DMN)] based on resting-state fMRI data (40, 41). The average BOLD time series were extracted separately across all ROIs regressing out confound signals (from fMRIPrep outputs). Confound regressors were loaded using nilearn. The denoising model included: 24 motion parameters (six rigid-body parameters, their temporal derivatives, and quadratic expansions), mean white matter and cerebrospinal fluid signals, and cosine basis regressors corresponding to high-pass filtering (cutoff = 0.01 Hz). The connectivity between each ROI pair was then calculated using the Pearson correlation coefficient. Correlation values were converted to z-values using Fisher’s r-z transformation to improve normality. A symmetric functional connectivity matrix of 400×400 size was created for each participant. Then, connectivity matrices of the participants in each group were averaged to obtain the average connectivity matrices of the depression group and the control group.

### 2.4 Gradient calculation

We used the BrainSpace Toolbox to calculate the gradients from the average connectivity matrices and individual matrices (42). Specifically; each column of the matrices was thresholded to preserve the strongest 10% functional connectivity values. This reduces the influence potentially noisy correlations and improves the stability of diffusion map embedding. A 10% threshold is commonly used in gradient analyses (3, 42). The similarity between all row pairs of each matrix was calculated with the normalized angle similarity coefficient, and positive symmetric affinity matrices reflecting the similarity of the connectivity profiles between each pair of regions were obtained. Then, non-linear diffusion map embedding technique was applied to these affinity matrices and the components (functional gradients) explaining the connectome variance were obtained in decreasing order. Functional gradients are represented as low-dimensional axes. The functional gradient scores associated with the regions represent their spatial locations in the embedding space. Thus, differences in the gradient scores of brain regions reflect their functional distances along the functional gradient axis. In order to ensure comparability between functional gradients obtained from both group matrices and functional gradients obtained from individual matrices of participants, gradient values ​​obtained from individual matrices and group matrices were aligned according to the template obtained from the average matrix of the healthy control group by Procrustes rotation (42). This step ensures that between-group comparisons reflect differences in gradient scores rather than arbitrary orientation differences. In this study, 10 gradients for the whole brain and 3 gradients for the insula were extracted. In line with previous studies (11, 21, 43), the focus was on the first gradient that explains the largest proportion of connectome variance (16% for MDD and %18 for whole brain, %42 for MDD and %41 for HC for insula-whole brain). Higher-order gradients account for progressively smaller variance and reflect more specific patterns of organization which was not in the scope of the present study.

#### 2.4.1 Calculation of insula gradients

In the Schaefer-Yeo atlas, 91 regions correspond to DMN, 47 regions to SN, 77 regions to SMN, 46 regions to DA, 52 regions to CN, 61 regions to VN, and 26 regions to LN. Within the SN regions, a total of 17 regions from both hemispheres (labels including “…SalVentAttn_FrOperIns…”) cover the insula regions. Insula regions, their MNI coordinates and corresponding labels in Schaefer Atlas are provided in **Supplementary Table 2**. Insula functional connectivity gradients were calculated based on the connectivity of the insula to seven brain networks. First, an insula-whole brain matrix (17x400) was extracted. Then, insula-brain network matrices were created representing the connectivity between each insula region and the regions in each brain network. Accordingly, seven insula-brain network matrices were obtained: insula-DMN (17x91), insula-SN (17x47), insula-SMN (17x77), insula-DAN (17x46), insula-CN (17x52), insula-VN (17x61), insula-LN (17x26). Then, the similarity between all row pairs of each matrix was calculated and eight (one for insula-whole brain and seven for insula-network) symmetric affinity matrices (17x17) were obtained, reflecting the similarity of the connectivity profiles between pairs of insula regions, associated with each brain network. Gradient calculations from the affinity matrices were made by following the same steps explained in whole-brain gradient calculations. Obtained insula gradients from individual matrices and group matrices were aligned to the average insula matrix of the healthy control group by Procrustes rotation.

### 2.5 Data analysis

For comparisons of sociodemographic data (age, years of education) and scale scores, IBM SPSS Statistics for Windows, Version 25.0 (Armonk, NY: IBM Corp) was used. For comparisons of whole brain gradient values between groups, an ANOVA was performed via MATLAB (version 2024a, MathWorks, USA), controlling for the effects of age and gender, and false discovery rate (FDR) correction was applied as a multiple comparison correction. For comparisons of insula-whole brain gradient values between groups, a two-way [2 (group: MDD, HC) x 17 (insula region: LH_FrOperIns1, …, RH_FrOperIns8)] mixed design ANOVA was performed via SPSS, controlling for the effects of age and gender, with group as the independent factor, and Bonferroni correction was applied as a multiple comparison correction. For the comparison of insula-seven brain network gradient values between groups, a three-way [2 (group: MDD, HC) x 7 (brain network: DMN, SN, DA, CN, VN, LN) x 17 (insula region: LH_FrOperIns1, …, RH_FrOperIns8)] mixed design ANOVA was performed using SPSS, with the group factor as the independent factor, controlling for the effects of age and gender, and Bonferroni correction was applied as a multiple comparison correction. Pearson correlation analysis was used to explore whether the regions showing significant differences between the groups were associated with the clinical scale scores.

**Figure 1:**
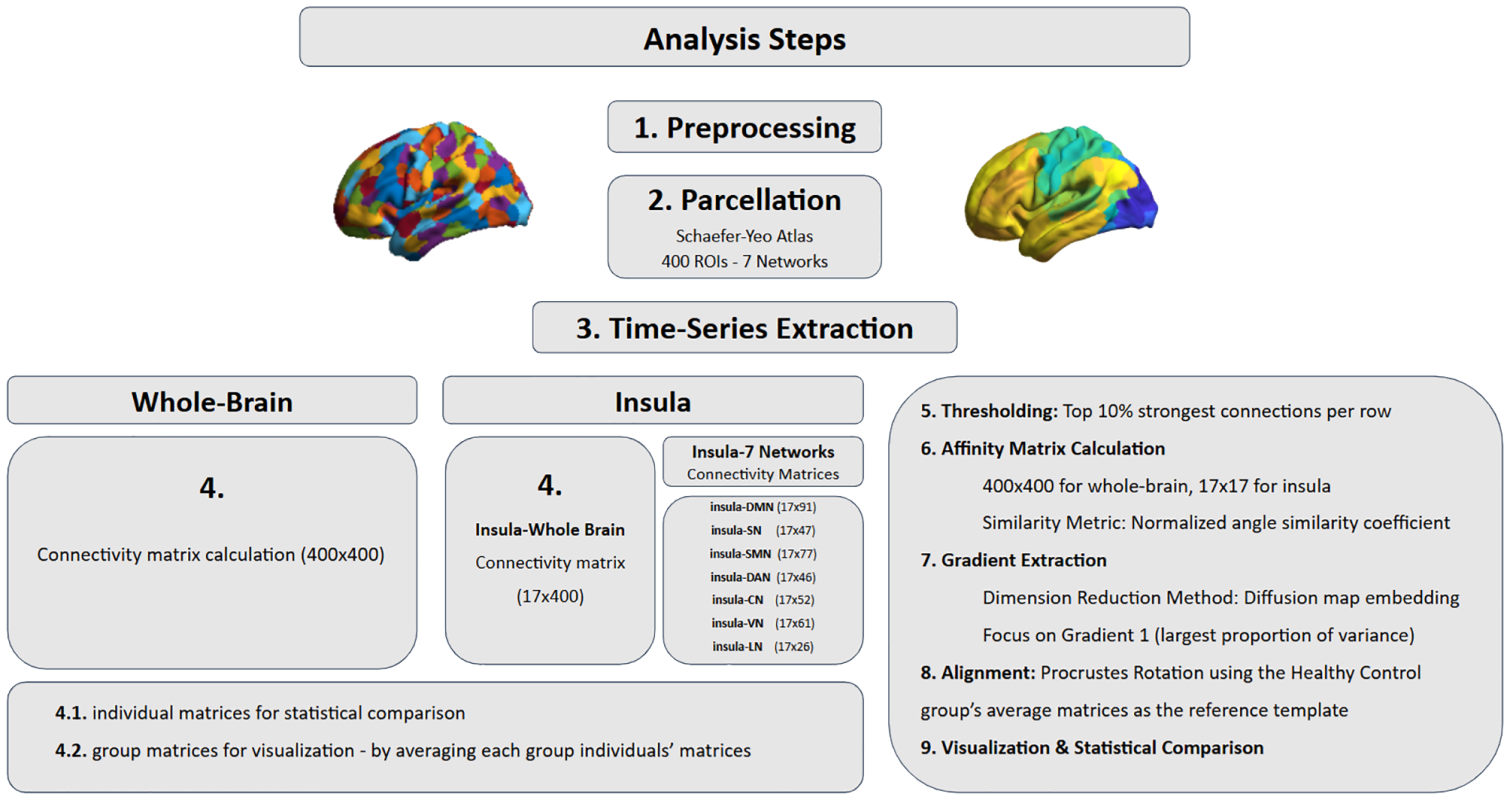
Data analysis steps. DMN, default mode network; SN, salience network; SMN, somatomotor network; DAN, dorsal attention network; CN, control network; VN, visual network; LN, limbic network.

Data analysis steps are shown in **Figure 1**.

## 3 Results

### 3.1 Demographic and clinical findings

There were no differences in age and years of education between groups (**Table 1**). HAM-D scores of the MDD group were significantly higher than the HC group as expected.

**Table 1:** Demographic and clinical variable comparisons of the MDD and HC group.

Variables	MDD (n=38)	HC (n=34)	*t*-value	*p*-value
Age	38.13 ± 11.46	37.12 ± 11.63	0.372	0.711
Years of education	11.94 ± 4.04	13.24 ± 3.99	-1.340	0.185
HAM-D Scores	26.71 ± 5.93	1.39 ± 1.46	25.44	<0.001

HAM-D, Hamilton Depression Assessment Scale; MDD, major depressive disorder; HC, healthy control.

### 3.2 Whole brain principal gradient findings

**Figure 2:**
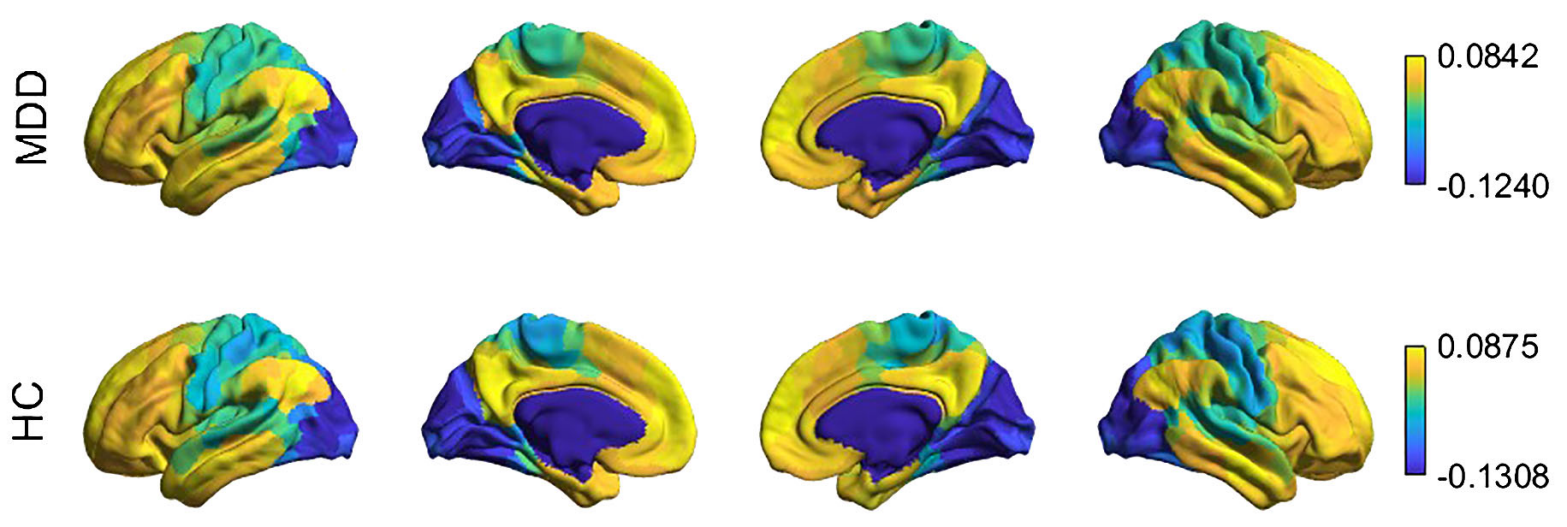
Whole brain principal gradients of the groups on the cortical surface. In both groups, transmodal areas (yellow), somatomotor (blue-green) and visual areas (dark blue) were located at separate points of the first gradient.

The first gradient explained similar proportions of variance, 16% in the depression group and 18% in the healthy control group, and separated VN and SMN areas from the DMN areas in both groups (**Figure 2**; **Supplementary Figures 1A**, **B**). Explained variance ratios (scaled eigenvalues) showed no significant difference between the groups (*t*(70) = -1.28, *p* = 0.205). In both groups, transmodal areas (yellow) and the unimodal sensory areas (blue, green) were located at opposite ends of the first gradient.

When the gradient scores of each brain region were compared between the groups, no significant differences were found (all *p*FDRs >.05).

The comparison of gradient value ranges (calculated as the difference between the gradient endpoints - largest and smallest gradient scores) revealed a more compressed distribution in the depression group (-0.1240 to 0.0842) compared to the control group, which exhibited a wider range (-0.1308 to 0.0875). However, this difference in gradient range was not statistically significant (*t*(70) = -1.043, *p* = 0.302).

**Figure 3:**
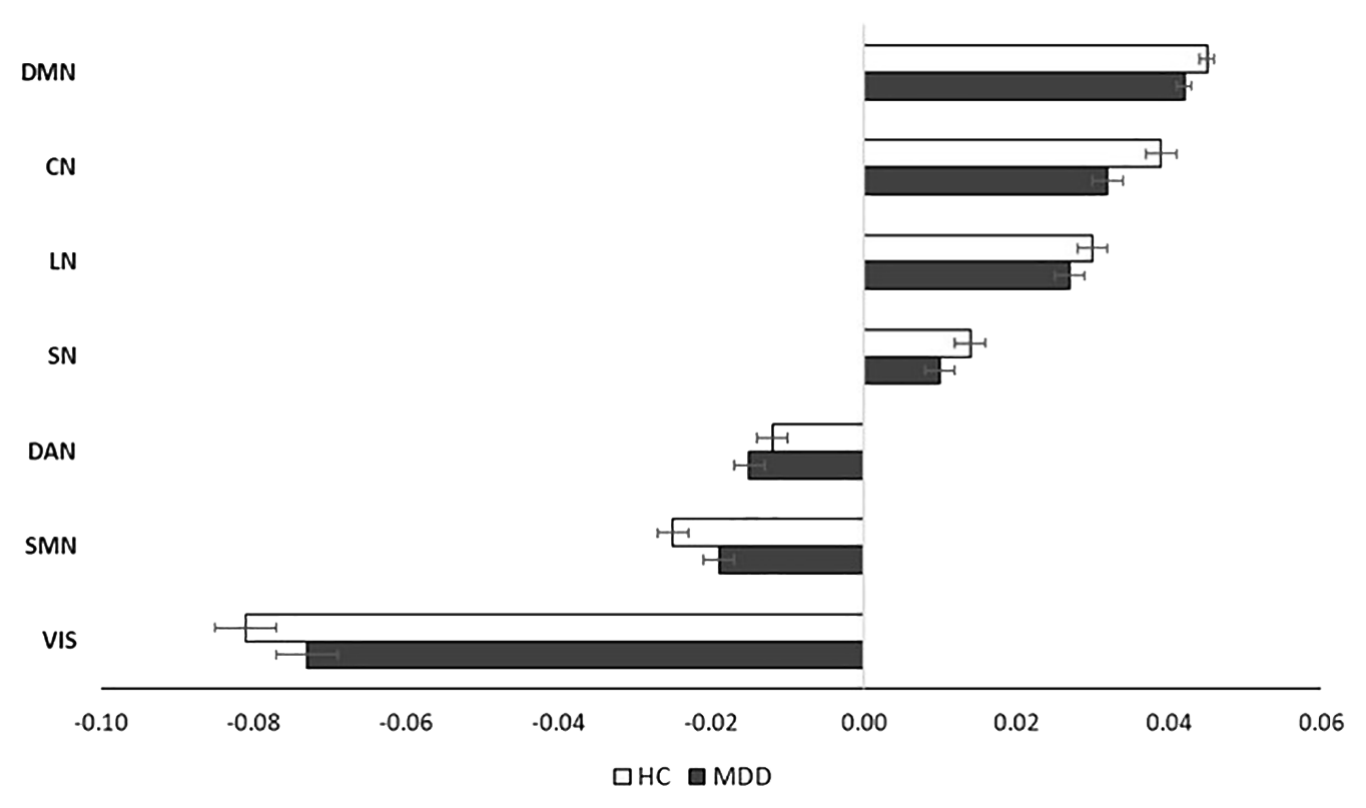
Average gradient scores of the seven brain networks of the groups (Error bars represent SEM). DMN, default mode network; SN, salience network; SMN, somatomotor network; DAN, dorsal attention network; CN, control network; VN, visual network; LN, limbic network.

**Figure 3** shows the corresponding parcels’ mean gradient scores for each functional network at the whole-brain level. No significant differences were observed between the groups (all *p*s>.05). The gradient range differences across the brain networks were not significant (all *p*s >.05).

### 3.3 Insula-to-whole brain principal gradient findings

The first gradient explained similar proportions of variance in both groups, explaining approximately 42% in the depression group (**Supplementary Figure 2A**) and 41% in the healthy control group (**Supplementary Figure 2B**). The variance ratios did not show significant difference between the groups (*t*(70) = 1.83, *p* = 0.072).

**Figure 4:**
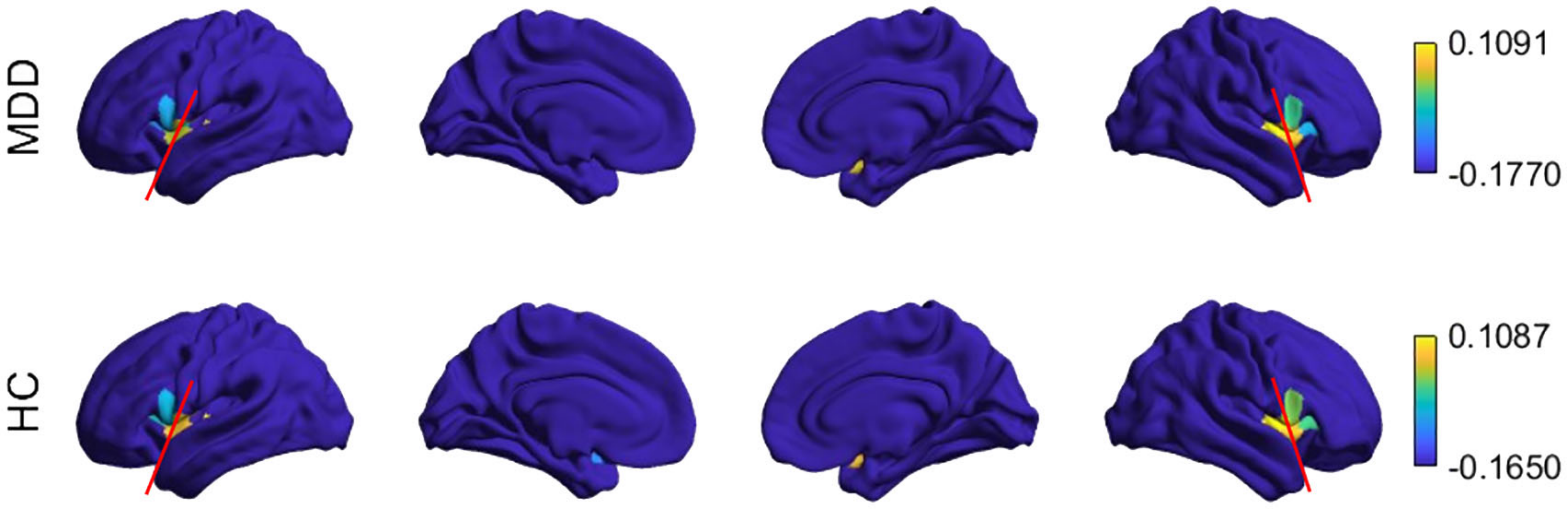
Insula-to-whole brain principal gradients of the groups on the cortical surface. The red reference line separates the anterior and posterior regions. The regions to the left and right of the red line are different colors in both groups, indicating that they fall at distant points of the gradient.

When examining the placement of insular regions along the gradient, the anterior and posterior areas were distinctly separated, positioned at distant points, and displayed different colors on the cortical surface (**Figure 4**). In the control group, the values of the dorsal anterior insula and posterior insula areas fell at the extremes of the gradient. However, in the depression group, both extremes corresponded to the dorsal anterior insula area.

The comparison of gradient ranges showed that the gradient values in the depression group were distributed over a wider range (-0.1770 to 0.1091), whereas the control group exhibited a more compressed range (-0.1650 to 0.1087). However, this difference in gradient range did not reach statistical significance (*t*(70) = 1.465, *p* = 0.147).

### 3.4 Insula-to-seven brain networks principal gradient findings

The proportions of variance explained by the insula-to-seven different brain network gradients were similar in both groups (all *p*s >.05, **Supplementary Table 1**).

**Figure 5:**
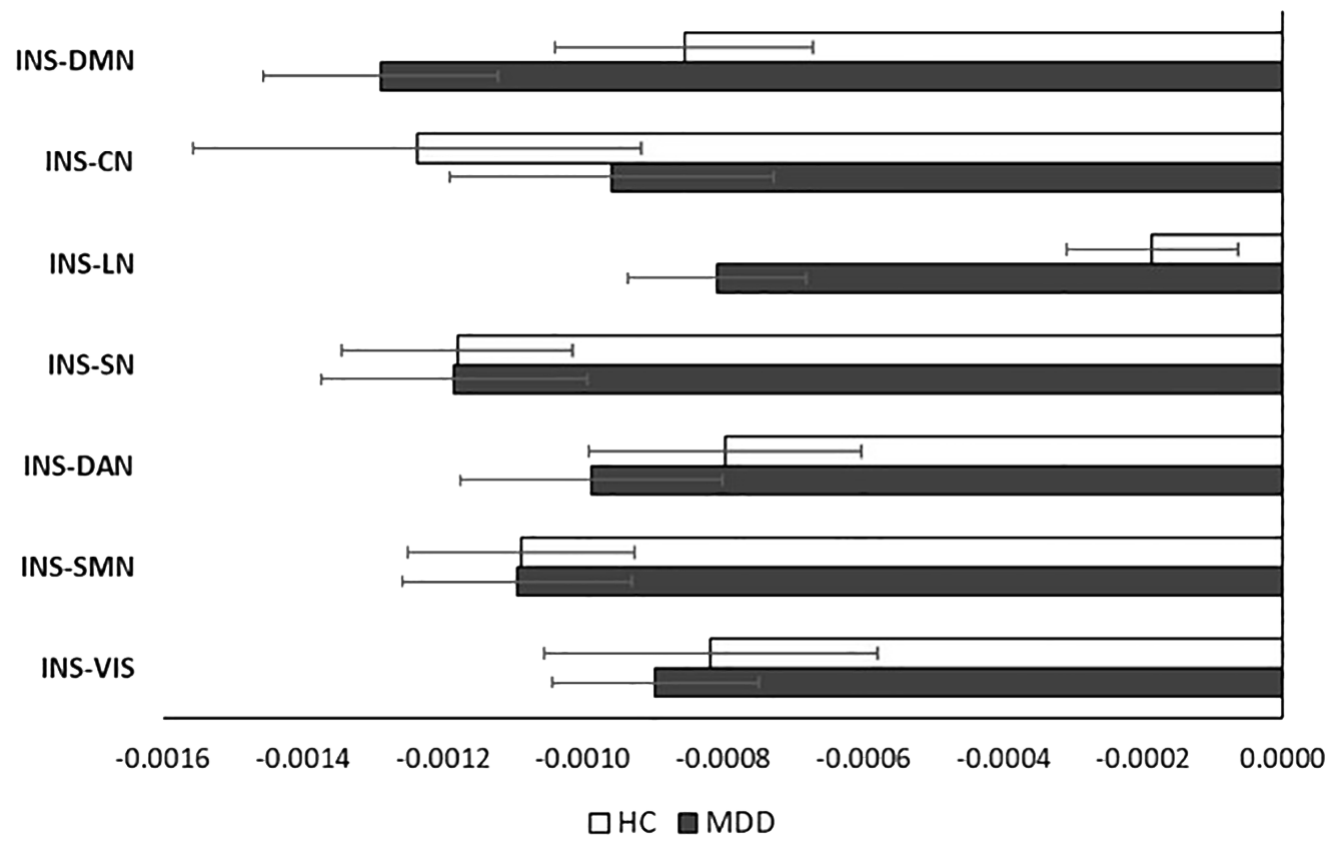
Average gradient scores representing insula functional connectivity to seven brain networks of the groups (Error bars represent SEM). DMN, default mode network; SN, salience network; SMN, somatomotor network; DAN, dorsal attention network; CN, control network; VN, visual network; LN, limbic network.

**Figure 5** shows the insula parcels’ mean gradient scores for each functional network. These means did not show any significant difference between the groups (all *p*s>.05). The gradient range differences of the brain networks were not significant (all *p*s >.05).

When examining which insula regions corresponded to the gradient endpoints, across the insula-to-DMN, CN, LN, SN, SMN, and VN gradients, both groups showed a similar organizational pattern: the gradient endpoints consistently corresponded to the dorsal anterior insula. In contrast, the insula-to-DAN gradient revealed a group-specific differentiation. In the depression group, the positive and negative gradient endpoints mapped onto the dorsal anterior insula (dAI) and ventral anterior insula (vAI), respectively. Whereas in the control group, both gradient extremes corresponded to the dAI.

**Figure 6:**
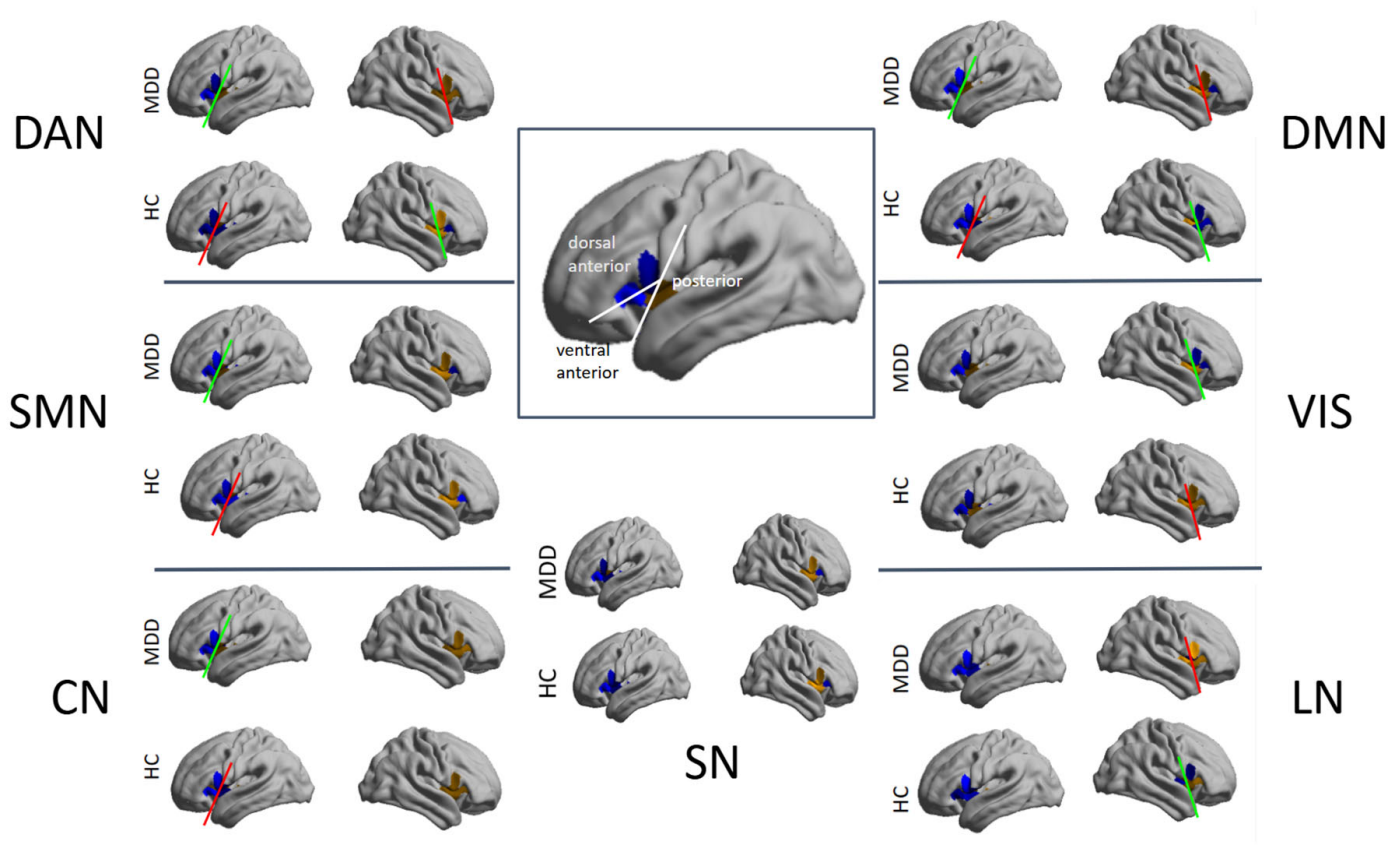
Insula-to-seven brain networks principal gradients of the groups on the cortical surface. The white reference lines on the brain figure placed inside the rectangle separate ventral anterior, dorsal anterior and posterior insular regions. A green line between anterior and posterior regions points to different coloring of the regions, indicating that they fall at distant points of the gradient. A red line points to no color difference of regions indicating that they fall at similar points of the gradient. For example, for SMN, anterior-posterior (blue-brown) regions were different colors in the MDD group, but not in the HC group (both blue).

However, visual inspection of cortical surface projections (**Figure 6**) revealed spatial distinctions that were not captured by the gradient endpoints. Except for the insula-SN gradient, on all network gradients, the anterior and posterior insula regions occupied separated positions (were different colors) along the gradients, even when they were not positioned at the endpoints. Importantly, these spatial configurations differed between groups. In the control group, the right anterior and right posterior insula regions were separated along the DAN, DMN, and LN gradients, whereas in the depression group these regions appeared closer together on these axes. Conversely, in the depression group, the left anterior and left posterior insula regions were separated along the DAN, SMN, DMN, and CON gradients, while they were positioned more closely in the control group. In the depression group, right anterior–posterior separation was preserved only along the VIS gradient.

The areas showing significant differences after the comparison of the gradient scores representing the relationship of each insula area with each brain network between the groups are given in **Table 2** below. The gradient scores of several dAI areas with DAN, DMN, SMN and SN, and the gradient of vAI area with DAN showed significant differences between the groups (*p*_corrected_ <. 05).

**Table 2:** The comparison of the gradient scores representing the relationship of each insula area with brain networks,.

Insula label	Network	Mean difference MDD-HC	p*	*F*	*η* ^2^	CI** (95%)
LH_SalVentAttn_FrOperIns_1 *(vAI)*	DAN	-0.042	0.043	4.262	0.059	-0.082, -0.001
LH_SalVentAttn_FrOperIns_3 *(dAI)*	DAN	-0.047	0.038	4.496	0.062	-0.090, -0.003
LH_SalVentAttn_FrOperIns_8 *(dAI-frontal operculum)*	DMN	0.041	0.007	7.599	0.101	0.011, 0.070
RH_SalVentAttn_FrOperIns_2 *(dAI)*	DMN	0.035	0.002	10.847	0.138	0.014, 0.056
RH_SalVentAttn_FrOperIns_6 *(dAI-frontal operculum)*	DMN	-0.043	0.017	6.030	0.081	-0.077, -0.008
RH_SalVentAttn_FrOperIns_7 *(dAI-frontal operculum)*	SMN	0.022	0.021	5.626	0.076	0.004, 0.041
LH_SalVentAttn_FrOperIns_8 *(dAI-frontal operculum)*	SN	0.038	0.026	5.175	0.071	0.005, 0.071

*Bonferroni-corrected. **Confidence Intervals. MDD, major depressive disorder; HC, healthy control; Vai, ventral anterior insula; dAI, dorsal anterior insula; DAN, dorsal attention network; DMN, default mode network; SMN, somatomotor network; SN, salience network.

### 3.5 The relationship between insula-to-seven brain network gradient values and HAM-D scores

**Figure 7:**
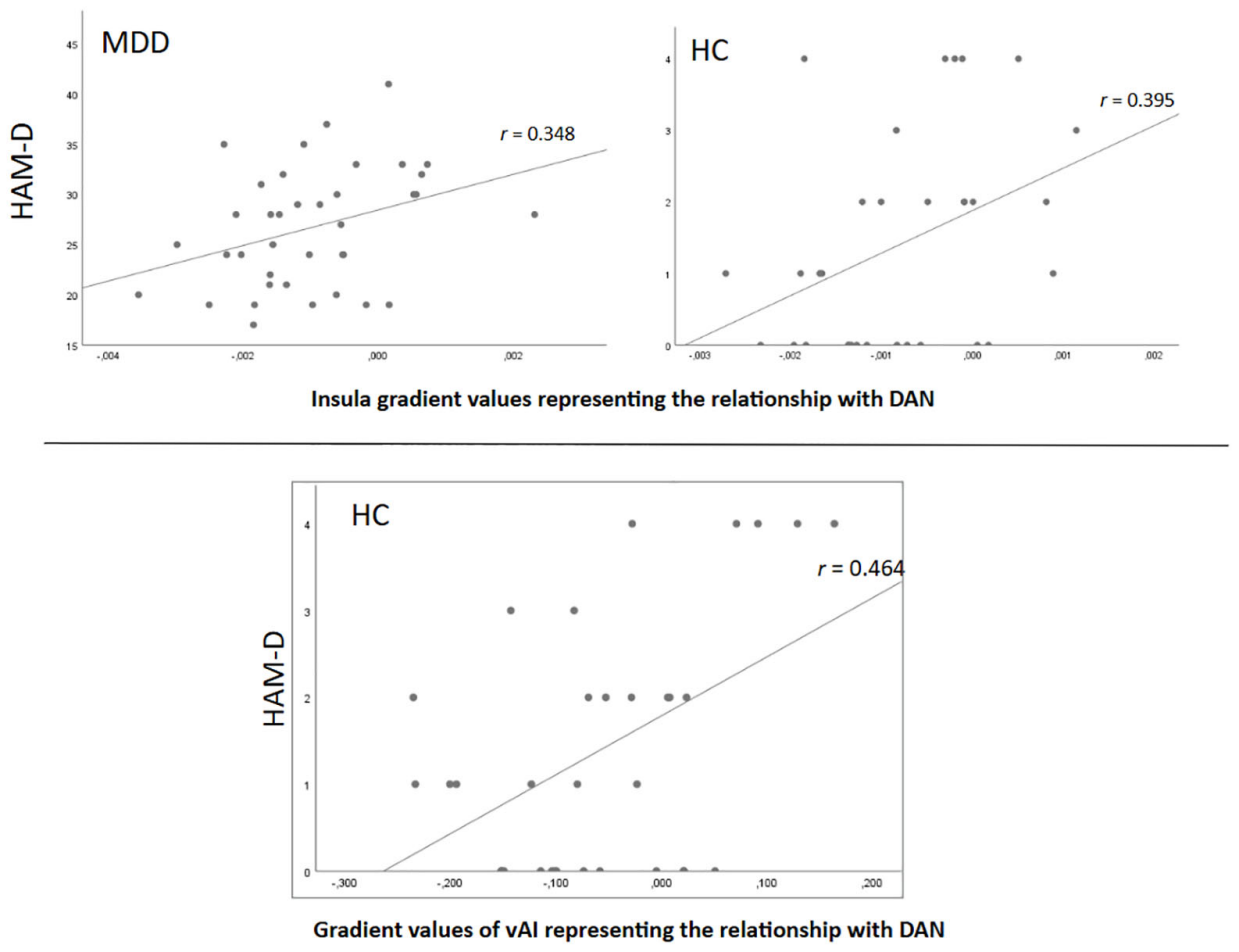
The relationship between insula-DAN gradient values and HAM-D scores.

The results showed a moderate positive correlation between the mean of the gradient values representing the relationship of the insula regions with DAN and the Hamilton Depression Scale scores in both the depression group (*p* = 0.032, *r* = 0.348) and the control group (*p* = 0.023, *r* = 0.395) (**Figure 7**, upper panel). Fisher’s r-to-z comparison indicated that the correlation between insula–DAN gradient values and HAM-D scores did not significantly differ between the MDD and control groups (z = −0.22, *p* = 0.412). Correlations (non-significant) between the mean of the gradient values representing the relationship of the insula regions with the other networks and the Hamilton Depression Scale scores are shown in **Supplementary Table 3**.

We also observed a moderate positive correlation between the gradient value representing the relationship of the LH_SalVentAttn_FrOperIns_1 (vAI) area with DAN and the HAM-D scores only in the control group (*p* = 0.007, *r* = 0.464) (**Figure 7**, lower panel). Correlations (non-significant) between other insula gradient values representing the relationship with brain networks and the HAM-D scores are shown in **Supplementary Table 4**.

## 4 Discussion

The present study examined the functional connectivity gradients of both the whole brain and the insula in depression and control groups. In accordance with the previous findings, the principal gradient separated transmodal regions from unimodal regions in both groups at the whole brain level. Insula-whole brain gradient separated the anterior and posterior insula areas less prominently in the depression group. The relationship of the insula regions with DAN among seven brain networks (DMN, SN, LN, CN, VN, SMN, DAN) were more specialized in depression. Ventral anterior and dorsal anterior insula areas had different gradient values in the MDD group for DMN, SMN, SN and especially for DAN, that is, some insula areas showed a differentiated connectivity profile with these networks compared to the healthy controls.

On the insula-whole brain gradient, both endpoints corresponded to anterior insula in the depression group while the control group’s gradient differentiated the anterior and posterior regions. This observation builds upon the previous work that showed anterior-posterior axis of the insula and highlights its disruption in depression (21, 37, 44). The anterior insula is a key region of the SN and for the interactions with other networks. It has a role in emotional awareness, interoception and cognition, while the posterior insula has a role in somatosensory processing (26, 27). Therefore, a diminished anterior-posterior differentiation may reflect dysregulation of hierarchical integration of bodily signals with higher-order emotional and cognitive evaluation, potentially contributing to depressive symptoms such as persistence of negative affect, fatigue, and difficulties in adaptive emotion regulation by transforming bodily states into subjective feeling states and maladaptive regulation responses (32).

Network-specific insula gradient comparisons showed that only the insula-to-DAN gradient separated insular areas, solely in the MDD group. The gradient endpoints corresponded to the dAI and vAI areas respectively in the depression group, while in the control group both ends corresponded to the dAI area, which suggests an increased functional differentiation between dorsal and ventral AI in depression. Considering DAN’s involvement in goal-directed attention processes, its interaction with the SN and its hypoconnectivity as a MDD-related neuropathology (30, 45, 46), this finding emphasizes the importance of differing insula-DAN connectivity in depression. Increased insula-DAN differentiation in depression might reflect maladaptive allocation of attention, such as attentional biases toward internal negative thoughts, and diminished ability to voluntarily shift attention away from negative stimuli or thoughts (47). This increased insula-DAN differentiation may also relate to altered interoceptive processing and emotion regulation in depression, given the insula’s role in integrating bodily signals with attentional systems that contribute to awareness and regulation of internal emotional states (26). Previously, reduced functional connectivity between insula and DAN in depression was shown (44) which was also associated with childhood maltreatment in individuals with MDD (48, 49). A study on the relationship between attention, early life stress and depression found an association between early life stress and within network connectivity of SN and DAN (50). Given the vAI’s role in socioemotional processes and the dAI’s role in cognition (27), and the link between childhood maltreatment and depression (51, 52), our results may be suggesting either the disruption of dorsal insula’s broad and necessary involvement during cognitive processes or a specialized engagement of ventral insula due to the emotional effects of past negative childhood experiences in depression. In addition, the average insula-DAN gradient positively correlated with HAM-D scores in both groups. More specifically, vAI-DAN gradient scores showed a significant difference between the groups and positively correlated with HAM-D scores in the HC group. The correlations between DAN gradients and symptom severity suggests that altered patterns have behavioral relevance. The presence of a significant positive correlation even within the HC group is interesting. One possible interpretation is that insula gradients capture variation in symptoms even within the non-clinical range. However, because the HAM-D scores in HC were low and had a restricted range, this interpretation should be considered exploratory, and future studies would be needed to determine whether insula-network gradients are sensitive to such small affective fluctuations.

Moreover, several insular regions’ gradient scores reflecting their relationship with networks differed between the groups. Lower gradient scores reflect a more similar functional connectivity profile with the regions on the negative end of the gradient (vAI for MDD), and higher gradient scores reflect a more similar functional connectivity profile with the regions on the positive end of the gradient (dAI for MDD). These findings suggest that in terms of their functional relationship similarity with DAN and DMN areas, insula regions with lower gradient scores had a more compatible profile with vAI’s profile in MDD compared to HC. And in terms of their functional relationship similarity with DMN, SMN and SN areas, insula regions with higher gradient scores had a more compatible profile with dAI’s profile in MDD compared to HC. The DMN gradient difference could reflect difficulties in disengaging from negative thoughts (53), the SMN gradient difference could reflect psychomotor symptoms of depression (54) and the SN gradient difference could reflect difficulties in detecting salient external stimuli and making proper emotional judgements (34). Overall, these network-specific alterations emphasize the importance of studying different insula areas’ connectivity patterns in depression.

Another observation from this study was the altered anterior-posterior and hemispheric distinctions between groups in certain networks which indicated network-dependent disruptions of insular functional organization. For example, for the right insula; the DAN, DMN and LN gradients separated the anterior and posterior regions in HC group but not in MDD group. For the left insula; the DAN, DMN, SMN and CN gradients separated the anterior and posterior regions in the MDD group but not in the HC group. This may suggest maladaptive reduced segregation of right insula subregions with DAN, DMN and LN and increased segregation of left insula subregions with DAN, DMN, SMN, CN in MDD. Previous research showed that pretreatment connectivity of right insula and DAN regions predicted depression (55), and linked decreased right insula connectivity with insufficient response to treatment (34). Right anterior insula has a role for modulating interactions between the DMN-CN and dysfunction in this interaction was associated with symptom severity (56). Taken together with our findings, these may point out to the potential importance of laterality of insula in depression (32).

We also would like to briefly mention our non-significant findings. The whole-brain principal gradient in both MDD and HC groups showed the unimodal-transmodal hierarchy, this was qualitative confirmation based on cortical surface visualization as commonly applied in gradient research. This served as an important validation step demonstrating that the extracted gradients reflected biologically meaningful large-scale organization. However, no quantitative group differences in gradient ranges or explained variance ratios were observed. Such null results could be informative in clarifying whether MDD is associated with global reorganization of cortical hierarchy or with region specific alterations. Our findings suggest preservation of global hierarchical structure, hinting at possible local hierarchical structures in line with our study’s aim. Still, significant differences in gradient range would have provided stronger evidence for altered hierarchical compression or expansion at the insula level. In the context of limited prior literature, we believe that even descriptive information regarding range distributions contributes to cumulative knowledge, so, we reported gradient ranges at both whole-brain and insula levels to allow readers to evaluate effect direction patterns.

To our knowledge, this is the first study to investigate functional connectivity gradients of the insula in MDD. Considering the heterogeneity of functional separation of insula subregions among individuals (37) and only a partial overlap between its gradients from different modalities (57) there was/is a need for more research to understand insula’s neurobiology. Several limitations of our study should be noted. First, our sample size was relatively small, which may limit statistical power. Although we applied multiple-comparison corrections (FDR and Bonferroni) to reduce the risk of false-positive findings, we did not conduct split-half reliability or bootstrapping analyses. Future studies with larger samples should use resampling-based methods to further test the robustness of the findings. Second, participants with depression had heterogeneous medication histories. While we applied stability criteria, medication effects cannot be completely ruled out. Third, our cross-sectional design prevents conclusions about causality or changes over time. In addition, our focus on the principal gradient for simplicity and comparability with previous studies may have overlooked potentially informative higher-order gradients. Whether the preservation of global hierarchical structure, but different local hierarchical patterns as found in this study is valid for insula and other important brain regions warrants further validation and exploration. If confirmed, such gradient features could be examined as potential predictors of symptom severity, diagnosis, or clinical outcome in longitudinal studies. In particular, insula-DAN gradient characteristics and insular hemispheric asymmetries (15) may hold promise as biomarkers of treatment response.

In conclusion, the findings represent an important extension of the existing literature on insular functional connectivity by capturing continuous organizational shifts rather than isolated pairwise connectivity differences. Our results showed that the anterior and posterior insula areas’ functional connectivity profiles with the whole brain differentiated less prominently in depression, but more prominently with DAN. These results were partially compatible and comparable with the existing literature on insula gradients to different networks (21). Therefore, our findings should be examined in larger, more clinically homogeneous samples and in longitudinal designs to assess their reproducibility and generalizability.

## Data Availability

The raw data supporting the conclusions of this article will be made available by the authors, without undue reservation.

## References

[1] World Health Organization . Depression and Other Common Mental Disorders: Global Health Estimates. Geneva: World Health Organization. (2017). Available online at: https://www.who.int/publications/i/item/depression-global-health-estimates.

[2] Huntenburg JM Bazin P-L Margulies DS . Large-scale gradients in human cortical organization. Trends Cognit Sci. (2018) 22:21–31. doi: 10.1016/j.tics.2017.11.002. PMID: 29203085

[3] Margulies DS Ghosh SS Goulas A Falkiewicz M Huntenburg JM Langs G . Situating the default-mode network along a principal gradient of macroscale cortical organization. Proc Natl Acad Sci USA. (2016) 113:12574–9. doi: 10.1073/pnas.1608282113. PMID: 27791099 PMC5098630

[4] Knodt AR Elliott ML Whitman ET Winn A Addae A Ireland D . Test–retest reliability and predictive utility of a macroscale principal functional connectivity gradient. Hum Brain Mapp. (2023) 44:6399–417. doi: 10.1002/hbm.26517. PMID: 37851700 PMC10681655

[5] Hong S-J Xu T Nikolaidis A Smallwood J Margulies DS Bernhardt B . Toward a connectivity gradient-based framework for reproducible biomarker discovery. NeuroImage. (2020) 223:117322. doi: 10.1016/j.neuroimage.2020.117322. PMID: 32882388

[6] Ruan L Chen G Yao M Li C Chen X Luo H . Brain functional gradient and structure features in adolescent and adult autism spectrum disorders. Hum Brain Mapp. (2024) 45:e26792. doi: 10.1002/hbm.26792. PMID: 39037170 PMC11261594

[7] Tian Y Zalesky A Bousman C Everall I Pantelis C . Insula functional connectivity in schizophrenia: Subregions, gradients, and symptoms. Biol Psychiatry Cognit Neurosci Neuroimaging. (2019) 4:399–408. doi: 10.1016/j.bpsc.2018.12.003. PMID: 30691966

[8] Pasquini L Fryer SL Eisendrath SJ Segal ZV Lee AJ Brown JA . Dysfunctional cortical gradient topography in treatment-resistant major depressive disorder. Biol Psychiatry Cognit Neurosci Neuroimaging. (2023) 8:928–39. doi: 10.1016/j.bpsc.2022.10.009. PMID: 36754677 PMC10150583

[9] Shiwei L Xiaojing Z Yingli Z Shengli C Xiaoshan L Ziyun X . Cortical hierarchy disorganization in major depressive disorder and its association with suicidality. Front Psychiatry. (2023) 14:1140915. doi: 10.3389/fpsyt.2023.1140915. PMID: 37168085 PMC10165114

[10] Wang J Zhou Y Ding J Xiao J . Functional gradient alteration in individuals with cognitive vulnerability to depression. J Psychiatr Res. (2021) 144:338–44. doi: 10.1016/j.jpsychires.2021.10.024. PMID: 34735837

[11] Wang X Xue L Hua L Shao J Yan R Yao Z . Structure-function coupling and hierarchy-specific antidepressant response in major depressive disorder. Psychol Med. (2024) 54:2688–97. doi: 10.1017/S0033291724000801. PMID: 38571298

[12] Xia M Liu J Mechelli A Sun X Ma Q Wang X . Connectome gradient dysfunction in major depression and its association with gene expression profiles and treatment outcomes. Mol Psychiatry. (2022) 27:1384–93. doi: 10.1038/s41380-022-01519-5. PMID: 35338312

[13] Xiao Y Wang D Tan Z Luo H Wang Y Pan C . Charting the dorsal-medial functional gradient of the default mode network in major depressive disorder. J Psychiatr Res. (2022) 153:1–10. doi: 10.1016/j.jpsychires.2022.06.059. PMID: 35792340

[14] Xiao Y Zhao L Zang X Xue S-W . Compressed primary-to-transmodal gradient is accompanied with subcortical alterations and linked to neurotransmitters and cellular signatures in major depressive disorder. Hum Brain Mapp. (2023) 44:5919–35. doi: 10.1002/hbm.26485. PMID: 37688552 PMC10619397

[15] Yang Y Zhen Y Wang X Liu L Zheng Y Zheng Z . Altered asymmetry of functional connectome gradients in major depressive disorder. Front Neurosci. (2024) 18:1385920. doi: 10.3389/fnins.2024.1385920. PMID: 38745933 PMC11092381

[16] Yin X Yang J Xiang Q Peng L Song J Liang S . Brain network hierarchy reorganization in subthreshold depression. NeuroImage Clin. (2024) 42:103594. doi: 10.1016/j.nicl.2024.103594. PMID: 38518552 PMC10973537

[17] Friedrich P Forkel SJ Thiebaut De Schotten M . Mapping the principal gradient onto the corpus callosum. NeuroImage. (2020) 223:117317. doi: 10.1016/j.neuroimage.2020.117317. PMID: 32882387 PMC7116113

[18] Jiang P Cui S Yao S Cai H Zhu J Yu Y . The hierarchical organization of the precuneus captured by functional gradients. Brain Struct Funct. (2023) 228:1561–72. doi: 10.1007/s00429-023-02672-5. PMID: 37378854 PMC10335959

[19] Shen Y Cai H Mo F Yao S Yu Y Zhu J . Functional connectivity gradients of the cingulate cortex. Commun Biol. (2023) 6:1–9. doi: 10.1038/s42003-023-05029-0. PMID: 37337086 PMC10279697

[20] Song Y Wang C Cai H Chen J Liu S Zhu J . Functional hierarchy of the angular gyrus and its underlying genetic architecture. Hum Brain Mapp. (2023) 44:2815–28. doi: 10.1002/hbm.26247. PMID: 36852603 PMC10089092

[21] Wang R Mo F Shen Y Song Y Cai H Zhu J . Functional connectivity gradients of the insula to different cerebral systems. Hum Brain Mapp. (2022) 44:790–800. doi: 10.1002/hbm.26099. PMID: 36206289 PMC9842882

[22] Yang S Meng Y Li J Li B Fan Y-S Chen H . The thalamic functional gradient and its relationship to structural basis and cognitive relevance. NeuroImage. (2020) 218:116960. doi: 10.1016/j.neuroimage.2020.116960. PMID: 32454205

[23] Zhang Q Zhang A Zhao Z Li Q Hu Y Huang X . Cognition-related connectome gradient dysfunctions of thalamus and basal ganglia in drug-naïve first-episode major depressive disorder. J Affect Disord. (2025) 370:249–59. doi: 10.1016/j.jad.2024.11.003. PMID: 39500466

[24] Menon V Uddin LQ . Saliency, switching, attention and control: a network model of insula function. Brain Struct Funct. (2010) 214:655–67. doi: 10.1007/s00429-010-0262-0. PMID: 20512370 PMC2899886

[25] Uddin LQ Nomi JS Hebert-Seropian B Ghaziri J Boucher O . Structure and function of the human insula. J Clin Neurophysiol Off Publ Am Electroencephalogr Soc. (2017) 34:300–6. doi: 10.1097/WNP.0000000000000377. PMID: 28644199 PMC6032992

[26] Laketić D Stojanović NM Laketić I Pavlović M Milosević B Starčević A . Insular cortex—biology and its role in psychiatric disorders: a narrative review. Brain Sci. (2025) 15:793. doi: 10.3390/brainsci15080793. PMID: 40867126 PMC12384716

[27] Kurth F Zilles K Fox P Laird A Eickhoff S . A link between the systems: functional differentiation and integration within the human insula revealed by meta-analysis. Brain Struct Funct. (2010) 214:519–34. doi: 10.1007/s00429-010-0255-z. PMID: 20512376 PMC4801482

[28] Fermin ASR Friston K Yamawaki S . An insula hierarchical network architecture for active interoceptive inference. R Soc Open Sci. (2022) 9:220226. doi: 10.1098/rsos.220226. PMID: 35774133 PMC9240682

[29] Berman MG Nee DE Casement M Kim HS Deldin P Kross E . Neural and behavioral effects of interference resolution in depression and rumination. Cognit Affect Behav Neurosci. (2011) 11:85–96. doi: 10.3758/s13415-010-0014-x. PMID: 21264648 PMC4006074

[30] Kaiser RH Andrews-Hanna JR Wager TD Pizzagalli DA . Large-scale network dysfunction in major depressive disorder: meta-analysis of resting-state functional connectivity. JAMA Psychiatry. (2015) 72:603–11. doi: 10.1001/jamapsychiatry.2015.0071. PMID: 25785575 PMC4456260

[31] Kaiser RH Whitfield-Gabrieli S Dillon DG Goer F Beltzer M Minkel J . Dynamic resting-state functional connectivity in major depression. Neuropsychopharmacol Off Publ Am Coll Neuropsychopharmacol. (2016) 41:1822–30. doi: 10.1038/npp.2015.352. PMID: 26632990 PMC4869051

[32] Sliz D Hayley S . Major depressive disorder and alterations in insular cortical activity: a review of current functional magnetic imaging research. Front Hum Neurosci. (2012) 6:323. doi: 10.3389/fnhum.2012.00323. PMID: 23227005 PMC3512092

[33] Fan J Tso IF Maixner DF Abagis T Hernandez-Garcia L Taylor SF . Segregation of salience network predicts treatment response of depression to repetitive transcranial magnetic stimulation. NeuroImage Clin. (2019) 22:101719. doi: 10.1016/j.nicl.2019.101719. PMID: 30776777 PMC6378906

[34] Geugies H Opmeer EM Marsman JBC Figueroa CA van Tol MJ Schmaal L . Decreased functional connectivity of the insula within the salience network as an indicator for prospective insufficient response to antidepressants. NeuroImage Clin. (2019) 24:102064. doi: 10.1016/j.nicl.2019.102064. PMID: 31795046 PMC6883326

[35] Li B-J Friston K Mody M Wang H-N Lu H-B Hu D-W . A brain network model for depression: from symptom understanding to disease intervention. CNS Neurosci Ther. (2018) 24:1004–19. doi: 10.1111/cns.12998. PMID: 29931740 PMC6490158

[36] Tura A Goya-Maldonado R . Brain connectivity in major depressive disorder: a precision component of treatment modalities? Transl Psychiatry. (2023) 13:1–17. doi: 10.1038/s41398-023-02499-y. PMID: 37296121 PMC10256697

[37] Tian Y Zalesky A . Characterizing the functional connectivity diversity of the insula cortex: subregions, diversity curves and behavior. NeuroImage. (2018) 183:716–33. doi: 10.1016/j.neuroimage.2018.08.055. PMID: 30172005

[38] Boré A Guay S Bedetti C Meisler S GuenTher N . Dcm2Bids (Version 3.1.1) [Computer software]. (2023). doi: 10.5281/zenodo.8436509. PMID:

[39] Esteban O Markiewicz CJ Blair RW Moodie CA Isik AI Erramuzpe A . fMRIPrep: a robust preprocessing pipeline for functional MRI. Nat Methods. (2019) 16:111–6. doi: 10.1038/s41592-018-0235-4. PMID: 30532080 PMC6319393

[40] Schaefer A Kong R Gordon EM Laumann TO Zuo X-N Holmes AJ . Local-global parcellation of the human cerebral cortex from intrinsic functional connectivity MRI. Cereb Cortex. (2018) 28:3095–114. doi: 10.1093/cercor/bhx179. PMID: 28981612 PMC6095216

[41] Yeo BTT Krienen FM Sepulcre J Sabuncu MR Lashkari D Hollinshead M . The organization of the human cerebral cortex estimated by intrinsic functional connectivity. J Neurophysiol. (2011) 106:1125–65. doi: 10.1152/jn.00338.2011. PMID: 21653723 PMC3174820

[42] Vos de Wael R Benkarim O Paquola C Lariviere S Royer J Tavakol S . BrainSpace: a toolbox for the analysis of macroscale gradients in neuroimaging and connectomics datasets. Commun Biol. (2020) 3:103. doi: 10.1038/s42003-020-0794-7. PMID: 32139786 PMC7058611

[43] Gonzalez Alam TJ Mckeown BLA Gao Z Bernhardt B Vos de Wael R Margulies DS . A tale of two gradients: differences between the left and right hemispheres predict semantic cognition. Brain Struct Funct. (2022) 227:631–54. doi: 10.1007/s00429-021-02374-w. PMID: 34510282 PMC8844158

[44] Peng X Lin P Wu X Gong R Yang R Wang J . Insular subdivisions functional connectivity dysfunction within major depressive disorder. J Affect Disord. (2018) 227:280–8. doi: 10.1016/j.jad.2017.11.018. PMID: 29128784

[45] Vossel S Geng JJ Fink GR . Dorsal and ventral attention systems: distinct neural circuits but collaborative roles. Neuroscientist. (2014) 20:150–9. doi: 10.1177/1073858413494269. PMID: 23835449 PMC4107817

[46] Gao Y Guo X Zhong Y Liu X Tian S Deng J . Decreased dorsal attention network homogeneity as a potential neuroimaging biomarker for major depressive disorder. J Affect Disord. (2023) 332:136–42. doi: 10.1016/j.jad.2023.03.080. PMID: 36990286

[47] Disner SG Beevers CG Haigh EAP Beck AT . Neural mechanisms of the cognitive model of depression. Nat Rev Neurosci. (2011) 12:467–77. doi: 10.1038/nrn3027. PMID: 21731066

[48] He C Fan D Liu X Wang Q Zhang H Zhang H . Insula network connectivity mediates the association between childhood maltreatment and depressive symptoms in major depressive disorder patients. Transl Psychiatry. (2022) 12:89. doi: 10.1038/s41398-022-01829-w. PMID: 35236833 PMC8891292

[49] Luo Q Chen J Li Y Wu Z Lin X Yao J . Aberrant brain connectivity is associated with childhood maltreatment in individuals with major depressive disorder. Brain Imaging Behav. (2022) 16:2021–36. doi: 10.1007/s11682-022-00672-3. PMID: 35906517

[50] Mao Y Xiao H Ding C Qiu J . The role of attention in the relationship between early life stress and depression. Sci Rep. (2020) 10:6154. doi: 10.1038/s41598-020-63351-7. PMID: 32273568 PMC7145865

[51] Gardner MJ Thomas HJ Erskine HE . The association between five forms of child maltreatment and depressive and anxiety disorders: A systematic review and meta-analysis. Child Abuse Negl. (2019) 96:104082. doi: 10.1016/j.chiabu.2019.104082. PMID: 31374447

[52] Nelson J Klumparendt A Doebler P Ehring T . Childhood maltreatment and characteristics of adult depression: Meta-analysis. Br J Psychiatry. (2017) 210:96–104. doi: 10.1192/bjp.bp.115.180752. PMID: 27908895

[53] Zhou H-X Chen X Shen Y-Q Li L Chen N-X Zhu Z-C . Rumination and the default mode network: Meta-analysis of brain imaging studies and implications for depression. NeuroImage. (2020) 206:116287. doi: 10.1016/j.neuroimage.2019.116287. PMID: 31655111

[54] Liang Q Xu Z Chen S Lin S Lin X Li Y . Temporal dysregulation of the somatomotor network in agitated depression. Brain Commun. (2024) 6:fcae425. doi: 10.1093/braincomms/fcae425. PMID: 39659972 PMC11630518

[55] Crowther A Smoski MJ Minkel J Moore T Gibbs D Petty C . Resting-state connectivity predictors of response to psychotherapy in major depressive disorder. Neuropsychopharmacology. (2015) 40:1659–73. doi: 10.1038/npp.2015.12. PMID: 25578796 PMC4915248

[56] Manoliu A Meng C Brandl F Doll A Tahmasian M Scherr M . Insular dysfunction within the salience network is associated with severity of symptoms and aberrant inter-network connectivity in major depressive disorder. Front Hum Neurosci. (2014) 7:930. doi: 10.3389/fnhum.2013.00930. PMID: 24478665 PMC3896989

[57] Royer J Paquola C Valk SL Kirschner M Hong S-J Park B . Gradients of brain organization: Smooth sailing from methods development to user community. Neuroinformatics. (2024) 22:623–34. doi: 10.1007/s12021-024-09660-y. PMID: 38568476

