# Supplementary material for: Functional connectivity gradients of the insula in major depressive disorder
Source: Front Psychiatry. 2026 Apr 15;17:1792843. doi: 10.3389/fpsyt.2026.1792843 (PMC13125075; doi:10.3389/fpsyt.2026.1792843)
Supplement: Supplementary file 1 [file DataSheet1.pdf]

## Data Preprocessing

Results included in this manuscript come from preprocessing performed using *fMRIPrep* 23.2.2 (@fmriprep1; @fmriprep2; RRID:SCR_016216), which is based on *Nipype* 1.8.6 (@nipype1; @nipype2; RRID:SCR_002502).

### *Anatomical data preprocessing*

A total of 1 T1-weighted (T1w) images were found within the input BIDS dataset. The T1w image was corrected for intensity non-uniformity (INU) with `N4BiasFieldCorrection` [@n4], distributed with ANTs 2.5.0 [@ants, RRID:SCR_004757], and used as T1w-reference throughout the workflow. The T1w-reference was then skull-stripped with a *Nipype* implementation of the `antsBrainExtraction.sh` workflow (from ANTs), using OASIS30ANTs as target template. Brain tissue segmentation of cerebrospinal fluid (CSF), white-matter (WM) and gray-matter (GM) was performed on the brain-extracted T1w using `fast` [FSL (version unknown), RRID:SCR_002823, @fsl_fast]. Volume-based spatial normalization to one standard space (MNI152NLin2009cAsym) was performed through nonlinear registration with `antsRegistration` (ANTs 2.5.0), using brain-extracted versions of both T1w reference and the T1w template. The following template was were selected for spatial normalization and accessed with *TemplateFlow* [23.1.0, @templateflow]: *ICBM 152 Nonlinear Asymmetrical template version 2009c* [@mni152nlin2009casym, RRID:SCR_008796; TemplateFlow ID: MNI152NLin2009cAsym].

### *Functional data preprocessing*

For each of the 1 BOLD runs found per subject (across all tasks and sessions), the following preprocessing was performed. First, a reference volume was generated, using a custom methodology of *fMRIPrep*, for use in head motion correction. Head-motion parameters with respect to the BOLD reference (transformation matrices, and six corresponding rotation and translation parameters) are estimated before any spatiotemporal filtering using `mcflirt` [FSL , @mcflirt]. The BOLD reference was then co-registered to the T1w reference using `mri_coreg` (FreeSurfer) followed by `flirt` [FSL , @flirt] with the boundary-based registration [@bbr] cost-function. Co-registration was configured with six degrees of freedom. Several confounding time-series were calculated based on the *preprocessed BOLD*: framewise displacement (FD), DVARS and three region-wise global signals. FD was computed using two formulations following Power (absolute sum of relative motions, @power_fd_dvars) and Jenkinson (relative root mean square displacement between affines, @mcflirt). FD and DVARS are calculated for each functional run, both using their implementations in *Nipype* [following the definitions by @power_fd_dvars]. The three global signals are extracted within the CSF, the WM, and the whole-brain masks. Additionally, a set of physiological regressors were extracted to allow for component-based noise correction [*CompCor*, @compcor]. Principal components are estimated after high-pass filtering the *preprocessed BOLD* time-series (using a discrete cosine filter with 128s cut-off) for the two *CompCor* variants: temporal (tCompCor) and anatomical (aCompCor). tCompCor components are then calculated from the top 2% variable voxels within the brain mask. For aCompCor, three probabilistic masks (CSF, WM and combined CSF+WM) are generated in anatomical space. The implementation differs from that of Behzadi et al. in that instead of eroding the masks by 2 pixels on BOLD space, a mask of pixels that likely contain a volume fraction of GM is subtracted from the aCompCor masks. This mask is obtained by thresholding the corresponding partial volume map at 0.05, and it ensures components are not extracted from voxels containing a minimal fraction of GM. Finally, these masks are resampled into BOLD space and binarized by thresholding at 0.99 (as

in the original implementation). Components are also calculated separately within the WM and CSF masks. For each CompCor decomposition, the *k* components with the largest singular values are retained, such that the retained components' time series are sufficient to explain 50 percent of variance across the nuisance mask (CSF, WM, combined, or temporal). The remaining components are dropped from consideration. The head-motion estimates calculated in the correction step were also placed within the corresponding confounds file. The confound time series derived from head motion estimates and global signals were expanded with the inclusion of temporal derivatives and quadratic terms for each [@confounds_satterthwaite_2013]. Frames that exceeded a threshold of 0.5 mm FD or 1.5 standardized DVARS were annotated as motion outliers. Additional nuisance timeseries are calculated by means of principal components analysis of the signal found within a thin band (*crown*) of voxels around the edge of the brain, as proposed by [@patriat_improved_2017]. All resamplings can be performed with *a single interpolation step* by composing all the pertinent transformations (i.e. head-motion transform matrices, susceptibility distortion correction when available, and co-registrations to anatomical and output spaces). Gridded (volumetric) resamplings were performed using `nitransforms`, configured with cubic B-spline interpolation.

Many internal operations of *fMRIPrep* use *Nilearn* 0.10.2 [@nilearn, RRID:SCR_001362], mostly within the functional processing workflow. For more details of the pipeline, see [the section corresponding to workflows in *fMRIPrep*'s documentation](https://fmriprep.readthedocs.io/en/latest/workflows.html "FMRIPrep's documentation").

### Copyright Waiver

The above boilerplate text was automatically generated by fMRIPrep with the express intention that users should copy and paste this text into their manuscripts *unchanged*.

It is released under the [CC0](https://creativecommons.org/publicdomain/zero/1.0/) license.

**Supplementary References**

Abraham, A., Pedregosa, F., Eickenberg, M., Gervais, P., Mueller, A., Kossaifi, J., et al. (2014). EnglishMachine learning for neuroimaging with scikit-learn. Frontiers in Neuroinformatics 8. doi:10.3389/fninf.2014.00014

Avants, B., Epstein, C., Grossman, M., and Gee, J. (2008). Symmetric diffeomorphic image registration with cross-correlation: Evaluating automated labeling of elderly and neurodegenerative brain. Medical Image Analysis 12, 26–41. doi:10.1016/j.media.2007.06.004

Behzadi, Y., Restom, K., Liau, J., and Liu, T. T. (2007). A component based noise correction method (CompCor) for BOLD and perfusion based fmri. NeuroImage 37, 90–101. doi:10.1016/j.neuroimage. 2007.04.042

Cox, R. W. and Hyde, J. S. (1997). Software tools for analysis and visualization of fmri data. NMR in Biomedicine 10, 171–178. doi:10.1002/(SICI)1099-1492(199706/08)10:4/5⟨171::AID-NBM453⟩3.0. CO;2-L

Esteban, O., Blair, R., Markiewicz, C. J., Berleant, S. L., Moodie, C., Ma, F., et al. (2018a). fmriprep. Software doi:10.5281/zenodo.852659

Esteban, O., Markiewicz, C., Blair, R. W., Moodie, C., Isik, A. I., Erramuzpe Aliaga, A., et al. (2018b). fMRIPrep: a robust preprocessing pipeline for functional MRI. Nature Methods doi:10. 1038/s41592-018-0235-4

Evans, A., Janke, A., Collins, D., and Baillet, S. (2012). Brain templates and atlases. NeuroImage 62, 911–922. doi:10.1016/j.neuroimage.2012.01.024

Fonov, V., Evans, A., McKinstry, R., Almli, C., and Collins, D. (2009). Unbiased nonlinear average age-appropriate brain templates from birth to adulthood. NeuroImage 47, Supplement 1, S102. doi:10. 1016/S1053-8119(09)70884-5

Gorgolewski, K., Burns, C. D., Madison, C., Clark, D., Halchenko, Y. O., Waskom, M. L., et al. (2011). Nipype: a flexible, lightweight and extensible neuroimaging data processing framework in python. Frontiers in Neuroinformatics 5, 13. doi:10.3389/fninf.2011.00013

Gorgolewski, K. J., Esteban, O., Markiewicz, C. J., Ziegler, E., Ellis, D. G., Notter, M. P., et al. (2018). Nipype. Software doi:10.5281/zenodo.596855

Greve, D. N. and Fischl, B. (2009). Accurate and robust brain image alignment using boundary-based registration. NeuroImage 48, 63–72. doi:10.1016/j.neuroimage.2009.06.060

Jenkinson, M., Bannister, P., Brady, M., and Smith, S. (2002). Improved optimization for the robust and accurate linear registration and motion correction of brain images. NeuroImage 17, 825–841. doi:10.1006/nimg.2002.1132

Jenkinson, M. and Smith, S. (2001). A global optimisation method for robust affine registration of brain images. Medical Image Analysis 5, 143–156. doi:10.1016/S1361-8415(01)00036-6

Lanczos, C. (1964). Evaluation of noisy data. Journal of the Society for Industrial and Applied Mathematics Series B Numerical Analysis 1, 76–85. doi:10.1137/0701007

Power, J. D., Mitra, A., Laumann, T. O., Snyder, A. Z., Schlaggar, B. L., and Petersen, S. E. (2014). Methods to detect, characterize, and remove motion artifact in resting state fmri. NeuroImage 84, 320–341. doi:10.1016/j.neuroimage.2013.08.048

Satterthwaite, T. D., Elliott, M. A., Gerraty, R. T., Ruparel, K., Loughead, J., Calkins, M. E., et al. (2013). An improved framework for confound regression and filtering for control of motion artifact in the preprocessing of resting-state functional connectivity data. NeuroImage 64, 240–256. doi:10.1016/j.neuroimage.2012.08.052

Tustison, N. J., Avants, B. B., Cook, P. A., Zheng, Y., Egan, A., Yushkevich, P. A., et al. (2010). N4itk: Improved n3 bias correction. IEEE Transactions on Medical Imaging 29, 1310–1320. doi:10.1109/TMI. 2010.2046908

Zhang, Y., Brady, M., and Smith, S. (2001). Segmentation of brain MR images through a hidden markov random field model and the expectation-maximization algorithm. IEEE Transactions on Medical Imaging 20, 45–57. doi:10.1109/42.906424

**Supplementary Figures**

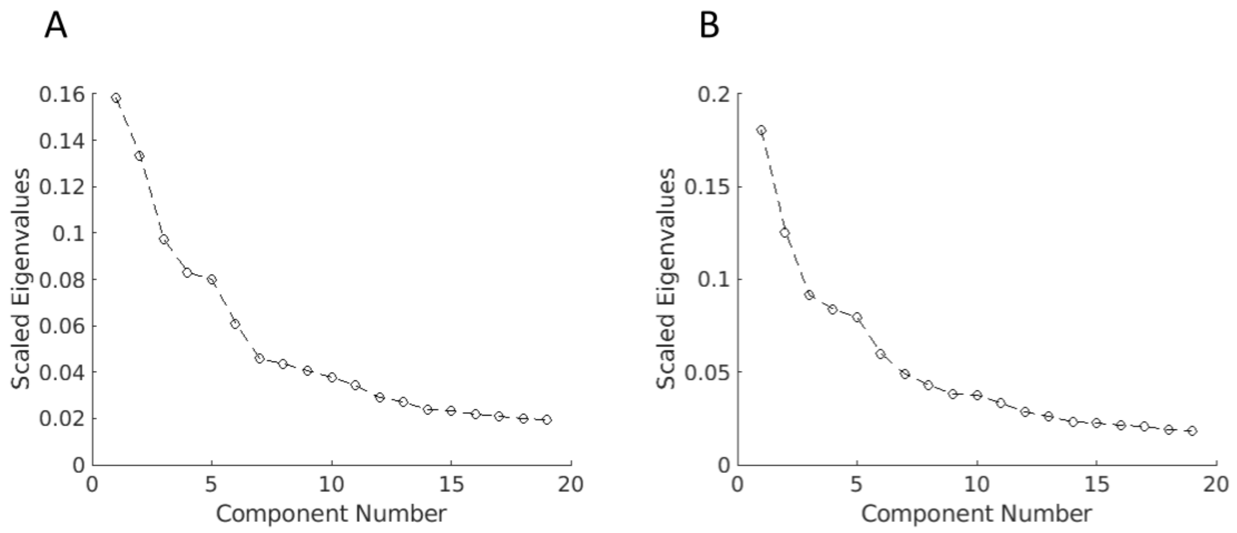


**Supplementary Figure 1.** Explained variances of the whole brain gradients of the groups

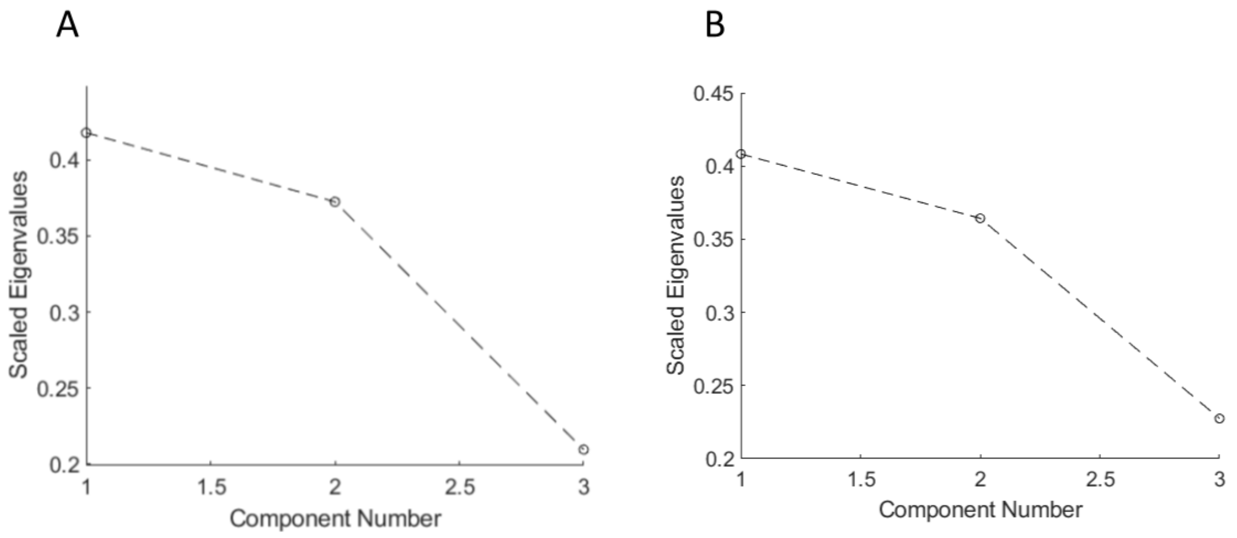


**Supplementary Figure 2.** Explained variances of the insula-to-whole brain gradients of the groups

**Supplementary Table 1**. The comparisons of the proportions of variance explained by the insula-to-seven different brain network gradients

| **Network** | **MDD** | **HC** | ***t*-value** | ***p*-value** |
|---|---|---|---|---|
| DMN | 43.42% | 42.67% | 0.072 | 0.443 |
| CN | 42.58% | 42.67% | -0.109 | 0.913 |
| DAN | 42.71% | 42.85% | -0.155 | 0.878 |
| LN | 43.97% | 44.40% | -0.361 | 0.719 |
| SN | 41.78% | 40.86% | 1.105 | 0.273 |
| SMN | 43.10% | 41.52% | 1.447 | 0.152 |
| VN | 42.23% | 41.57% | 0.679 | 0.499 |

MDD: major depressive disorder, HC: healthy control, DMN: default mode network, CN: control network, DAN: dorsal attention network, LN: limbic network, SN: salience network, SMN: somatomotor network, VN: visual network

**Supplementary Table 2.** Insula regions & MNI Coordinates in Schaefer Atlas and their corresponding labels

| ROI Number & Name | | Label | MNI Coordinates | | |
|---|---|---|---|---|---|
| | | | x | y | z |
| 97 | 7Networks_LH_SalVentAttn_FrOperIns_1 | vAI | -44 | 5 | -17 |
| 98 | 7Networks_LH_SalVentAttn_FrOperIns_2 | dAI | -39 | 2 | -4 |
| 99 | 7Networks_LH_SalVentAttn_FrOperIns_3 | dAI | -33 | 25 | -1 |
| 100 | 7Networks_LH_SalVentAttn_FrOperIns_4 | pI | -40 | -15 | -2 |
| 101 | 7Networks_LH_SalVentAttn_FrOperIns_5 | dAI | -33 | 19 | 8 |
| 102 | 7Networks_LH_SalVentAttn_FrOperIns_6 | dAI | -36 | 4 | 11 |
| 103 | 7Networks_LH_SalVentAttn_FrOperIns_7 | dAI | -43 | 12 | 2 |
| 104 | 7Networks_LH_SalVentAttn_FrOperIns_8 | dAI-frontal operculum | -50 | 1 | 5 |
| 105 | 7Networks_LH_SalVentAttn_FrOperIns_9 | dAI-frontal operculum | -52 | 9 | 13 |
| 302 | 7Networks_RH_SalVentAttn_FrOperIns_1 | vAI | 40 | 5 | -15 |
| 303 | 7Networks_RH_SalVentAttn_FrOperIns_2 | dAI | 41 | 8 | -3 |
| 304 | 7Networks_RH_SalVentAttn_FrOperIns_3 | pI | 40 | -10 | -4 |
| 305 | 7Networks_RH_SalVentAttn_FrOperIns_4 | pI | 39 | -2 | 6 |
| 306 | 7Networks_RH_SalVentAttn_FrOperIns_5 | dAI | 37 | 23 | 5 |
| 307 | 7Networks_RH_SalVentAttn_FrOperIns_6 | dAI-frontal operculum | 38 | 7 | 11 |
| 308 | 7Networks_RH_SalVentAttn_FrOperIns_7 | dAI-frontal operculum | 49 | 5 | 3 |
| 309 | 7Networks_RH_SalVentAttn_FrOperIns_8 | dAI-frontal operculum | 54 | 12 | 12 |

vAI: ventral anterior insula, dAI: dorsal anterior insula, pI: posterior insula

**Supplementary Table 3.** Correlations between the mean of the gradient values representing the relationship of the insula regions with seven brain networks and the HAM-D scores

| | | | DAN | DMN | SMN | VIS | SAL | LN | CON |
|---|---|---|---|---|---|---|---|---|---|
| **MDD** | **HAM-D** | *r* | 0.348 | -0.061 | -0.066 | 0.054 | -0.098 | 0.080 | 0.108 |
| | | *p* | 0.032* | 0.730 | 0.710 | 0.762 | 0.582 | 0.652 | 0.542 |
| **HC** | | *r* | 0.395 | -0.024 | -0.187 | 0.336 | -0.112 | -0.062 | 0.300 |
| | | *p* | 0.023* | 0.899 | 0.315 | 0.065 | 0.549 | 0.741 | 0.102 |

MDD: major depressive disorder, HC: healthy control, HAM-D: Hamilton Depression Scale, DMN: default mode network, CN: control network, DAN: dorsal attention network, LN: limbic network, SN: salience network, SMN: somatomotor network, VN: visual network

**Supplementary Table 4.** Correlations between insula-network gradient values that exhibited significant differences and the HAM-D scores

| | | **MDD** | | **HC** | |
|---|---|---|---|---|---|
| | | **Hamilton Depression Scale** | | | |
| **Insula Label** | **Network** | *r* | *p* | *r* | *p* |
| LH_SalVentAttn_FrOperIns_1 *(vAI)* | DAN | -0.061 | 0.717 | 0.464 | 0.007* |
| LH_SalVentAttn_FrOperIns_3 *(dAI)* | DAN | 0.159 | 0.370 | -0.205 | 0.268 |
| LH_SalVentAttn_FrOperIns_8 *(dAI-frontal operculum)* | DMN | -0.173 | 0.327 | -0.008 | 0.967 |
| RH_SalVentAttn_FrOperIns_2 *(dAI)* | DMN | 0.174 | 0.324 | 0.435 | 0.014 |
| RH_SalVentAttn_FrOperIns_6 *(dAI-frontal operculum)* | DMN | -0.131 | 0.461 | -0.196 | 0.292 |
| RH_SalVentAttn_FrOperIns_7 *(dAI-frontal operculum)* | SMN | 0.449 | 0.080 | 0.049 | 0.793 |
| LH_SalVentAttn_FrOperIns_8 *(dAI-frontal operculum)* | SN | -0.169 | 0.340 | 0.345 | 0.058 |

MDD: major depressive disorder, HC: healthy control, vAI: ventral anterior insula, dAI: dorsal anterior insula, *p < 0.05.
